# Rational design peptide inhibitors of Cyclophilin D as a potential treatment for acute pancreatitis

**DOI:** 10.1097/MD.0000000000036188

**Published:** 2023-12-01

**Authors:** Yuehong Li, Ting Liu, Xiaoyan Lai, Huifang Xie, Heng Tang, Shuangchan Wu, Yongshun Li

**Affiliations:** a Department of Critical Care Medicine, Longyan First Affiliated Hospital of Fujian Medical University, Longyan, China; b Key Laboratory of Bioorganic Synthesis of Zhejiang Province, College of Biotechnology and Bioengineering, Zhejiang University of Technology, Hangzhou, China; c Institute of Medical Research, Northwestern Polytechnical University, Xian, Shanxi Province, China.

**Keywords:** acute pancreatitis, Cyclophilin D, molecular docking, molecular dynamics simulation, peptide inhibitor

## Abstract

Cyclophilin D (CypD) is a mitochondrial matrix peptidyl prolidase that regulates the mitochondrial permeability transition pore. Inhibition of CypD was suggested as a therapeutic strategy for acute pancreatitis. Peptide inhibitors emerged as novel binding ligand for blocking receptor activity. In this study, we present our computational approach for designing peptide inhibitors of CypD. The 3-D structure of random peptides were built, and docked into the active center of CypD using Rosetta script integrated FlexPepDock module. The peptide displayed the lowest binding energy against CypD was further selected for virtual iterative mutation based on virtual mutagenesis and molecular docking. Finally, the top 5 peptides with the lowest binding energy was selected for validating their affinity against CypD using inhibitory assay. We showed 4 out of the selected 5 peptides were capable for blocking the activity of CypD, while WACLQ display the strongest affinity against CypD, which reached 0.28 mM. The binding mechanism between WACLQ and CypD was characterized using molecular dynamics simulation. Here, we proved our approach can be a robust method for screening peptide inhibitors.

## 1. Introduction

The mitochondrial permeability transition pore (mPTP) is a nonselective channel located in the inner mitochondrial membrane. It plays a crucial role in maintaining mitochondrial homeostasis, regulating cell death pathways, and controlling mitochondrial function.^[1–3]^ When the mPTP opens excessively, it leads to mitochondrial dysfunction, oxidative stress, and cell death.^[4,5]^ Acute pancreatitis is an inflammatory condition characterized by the sudden inflammation of the pancreas.^[6]^ It can range from a mild, self-limiting condition to a severe, life-threatening illness. The pathogenesis of acute pancreatitis (AP) involves various factors, including premature activation of digestive enzymes within the pancreas, inflammation, and local and systemic complications.^[7]^ The opening of the mPTP in pancreatic acinar cells allows the release of calcium ions (Ca^2+^) from the mitochondrial matrix into the cytosol, the increased Ca^2+^ in cytosolic triggers further cell injury and inflammatory responses.^[8]^ Additionally, the opening of the mPTP results in the collapse of the mitochondrial membrane potential, impairment of oxidative phosphorylation, and the release of pro-apoptotic factors from the mitochondria.^[9]^ These events contribute to cell death and exacerbate the severity of AP.

Cyclophilin D (CypD) is specifically located within the mitochondrial matrix and is an important modulator of mPTP opening.^[10–12]^ When CypD interacts with other proteins in the mitochondrial membrane, it promotes the opening of the mPTP.^[13]^ This interaction is particularly influenced by the level of Ca^2+^ in the mitochondria. CypD acts as a positive regulator of mPTP, facilitating its opening in response to different stimuli. Modulating CypD activity or inhibiting mPTP opening has shown promise in preclinical studies as a potential therapeutic strategy to protect mitochondria and mitigate cellular damage in these conditions.^[14,15]^ Inhibiting CypD is a potential strategy for modulating the activity of the mPTP and protecting against mitochondrial dysfunction and cell death. Previously, small molecules and compounds have been developed to directly inhibit the activity of CypD.^[16]^ These inhibitors can bind to CypD to disrupt its interaction with other proteins involved in mPTP opening. Currently, CypD inhibitors have emerged as a potential therapeutic approach for the treatment of AP.^[17]^

Peptide drugs have gained significant attention in the field of therapeutics, which were adopted for the treatment of various diseases.^[18]^ Peptide drugs exhibit high specificity against their targets due to their ability to interact with specific receptors or proteins, which can minimize off-target effects and enhance therapeutic efficacy.^[19]^ Peptides derived from naturally occurring proteins can mimic endogenous molecules, which display great biocompatibility and reduced toxicity compared to synthetic drugs.^[20]^ Moreover, peptides are more likely to be biodegradable, nontoxic, and less prone to accumulate in tissues compared to some small molecule drugs. Growing attention has been paid to rational design of peptide drugs to decrease the experimental costs.^[21–23]^ Recently, the rapid development of structural biology has led to the discovery of protein or peptide drugs entering a new era.^[24]^ Adopting Rosetta for protein and peptide design has achieved great success.^[25]^ Several methods were introduced for the design of peptide drugs. For the top-down process, a peptide library was built to dock into the receptor active pocket.^[26–28]^ While for the bottom-up method, the binding sites were designed within the peptide prioritize to peptide assembling.

Both peptides and small molecules were previously shown their ability for blocking the activity of CypD.^[29]^ In this study, we introduced a top-down process for designing peptide binders of CypD. We developed a Python script, and integrated Rosetta modules for building peptide library and perform molecular docking. The random peptides were built their structure using Rosetta BuildPeptide module. The peptides were then accommodated into the active center of CypD to perform molecular docking. We selected the peptides with the lowest binding energy against CypD for virtual iterative mutagenesis. We finally characterized 5 peptides with the lowest binding energy against CypD using inhibitory assay. Molecular dynamics (MD) simulation was carried out to understand the binding mechanism between peptide and protein.

## 2. Materials and methods

### 2.1. Preparation of CypD as docking receptor

The crystal structure of CypD bound to its inhibitor (PDB ID: 6R8O) was selected as docking receptor in this study.^[30]^ This crystal structure with a high resolution of 1.36 Å. Water molecule and the original inhibitor were removed from this structure, and the structure was refined using 2 rounds of Rosetta Relax.^[31]^ In the first round, protein side chains were repacked, while the second round the whole protein structure was fully minimized (command line in Table S1, Supplemental Digital Content, http://links.lww.com/MD/K884). We used Rosetta ref2015 for scoring protein,^[32]^ while the structure with the lowest Rosetta score was selected for the next step during each round of relax.

### 2.2. Generating peptide library

In order to create peptide candidates for docking, we used Python script to generate random peptide sequences. The 3-D structure of these random peptides were built using Rosetta BuildPeptide.^[33]^ Due to short peptides can be either high flexible or rigidity, the peptide conformation was captured by simulating its backbone using Monte Carlo simulation integrated in Rosetta script, which named CartesianMD (command line in Table S1, Supplemental Digital Content, http://links.lww.com/MD/K884). Noted that peptides with conformational refinement was then subjected to molecular docking.

### 2.3. Molecular docking

**Figure 1.**
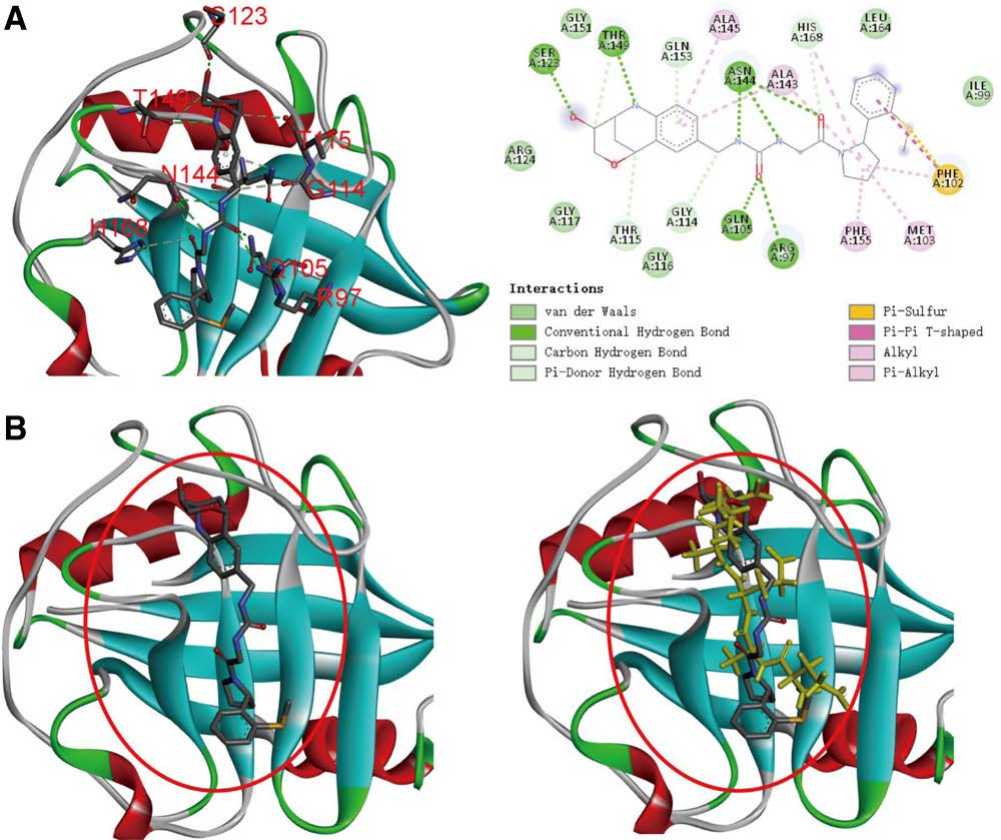
The interaction between CypD and its binding partner. (1) Binding structure of CypD against its binding partner was from PDB ID: 6R8O; (B) accommodating peptide into the binding pocket of CypD according to the position of its native binding partner.

**Figure 2.**
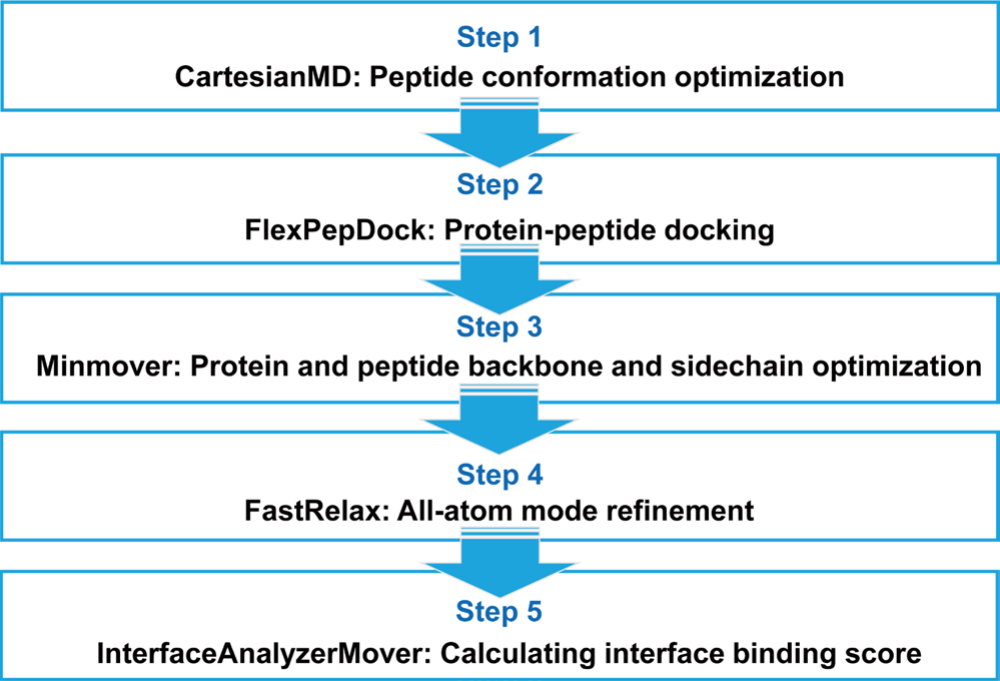
The protocol for protein-peptide molecular docking.

Molecular docking was carried out relied on Rosetta script.^[33]^ To perform docking, the docking pocket was selected based on the co-crystal structure as shown in Figure 1A. The peptide was accommodated into the docking pocket according to the original binding partner using Gromacs-2020 editconf^[34]^ (Fig. 1B). Peptide conformation optimization was firstly performed, and the Rosetta script for molecular docking was integrated as follows (Fig. 2): (1) FlexPepDock mover for optimizing the interactions between protein and peptide^[35]^; (2) optimizing the backbone and sidechains using Minmover; (3) perform all-atoms refinement using FastRelax; (4) calculating interface binding energy using InterfaceAnalyzerMover. (All relevant command in Table S2, Supplemental Digital Content, http://links.lww.com/MD/K885.) Noted that the molecular docking in this study carried out by simulating the interactions between the docked peptide and CypD.

### 2.4. Virtual iteration of peptides

To discover the peptide with the strongest affinity against CypD, we used the peptide with the lowest protein–peptide Rosetta score for iteration. Single amino acid insertion was introduced to the peptide to generate another peptide library (command line in Table S2, Supplemental Digital Content, http://links.lww.com/MD/K885). These novel generated peptides were docked into CypD according to the protocol described above. We selected the peptides with the lowest score for experimental validation.

### 2.5. Molecular dynamics simulation

MD simulation was carried out to understand the binding mechanism between CypD and the docked peptides. The docking complex obtained from molecular docking was used to perform MD simulation using Gromacs-2020.^[34]^ The simulation system was filled with SPC/E water in a cubic box, and neutralized using Na^+^ and Cl^−^. We used 15 Å as the distance between the surface of the docking complex and the edge of the water box. We adopted the steepest descent method for initial energy minimization, and the system was equilibrated using the isochoric–isothermal ensemble and isothermal–isovolumetric ensemble under 300 K for 100 and 200 ps, respectively. The MD simulation was carried out for 100 ns under 300 K. The critical sites for protein–peptide binding was revealed using gmxMMPBSA.^[36]^ The last 10 ns of simulation was extracted for gmxMMPBSA analysis.

### 2.6. The inhibitory assay

The method for measuring half-maximal inhibitory concentration (IC_50_) values of the peptides against CypD was described previously as chymotrypsin coupled prolyl-isomerase assay. Human recombinant CypD was purchased from Beyotime (Shanghai, China). The substrate solution for inhibitory assay was prepared as 100 μM Suc-AAPF-AMC (Chymotrypsin Substrate II, Sigma–Aldrich, Shanghai, China) with varied concentration of selected peptide (varied from 0.001 to 100 μM), 15 μg/mL CypD, and 0.5 M LiCl/2,2,2-trifluoroethanol. Alpha-chymotrypsin from bovine pancreas was purchased from Sigma (Shanghai, China) and dissolved in 0.001 M HCl, the concentration for chymotrypsin was prepared to 5 mg/mL. For inhibitory assay, the 96-well plate was filled with 150 μL of the substrate solution and incubated at 25 °C for 1 hour, followed by adding 40 μL of the chymotrypsin solution. The fluorescent changes under 380 nm was captured every 5 seconds for 300 seconds using Mltrospec 3100. The IC_50_ value was calculated which indicated the peptide concentration needed for obtaining 50% of thrombin inhibitory activity.

## 3. Results and discussion

### 3.1. Simulating 3-D conformation of random peptides

**Figure 3.**
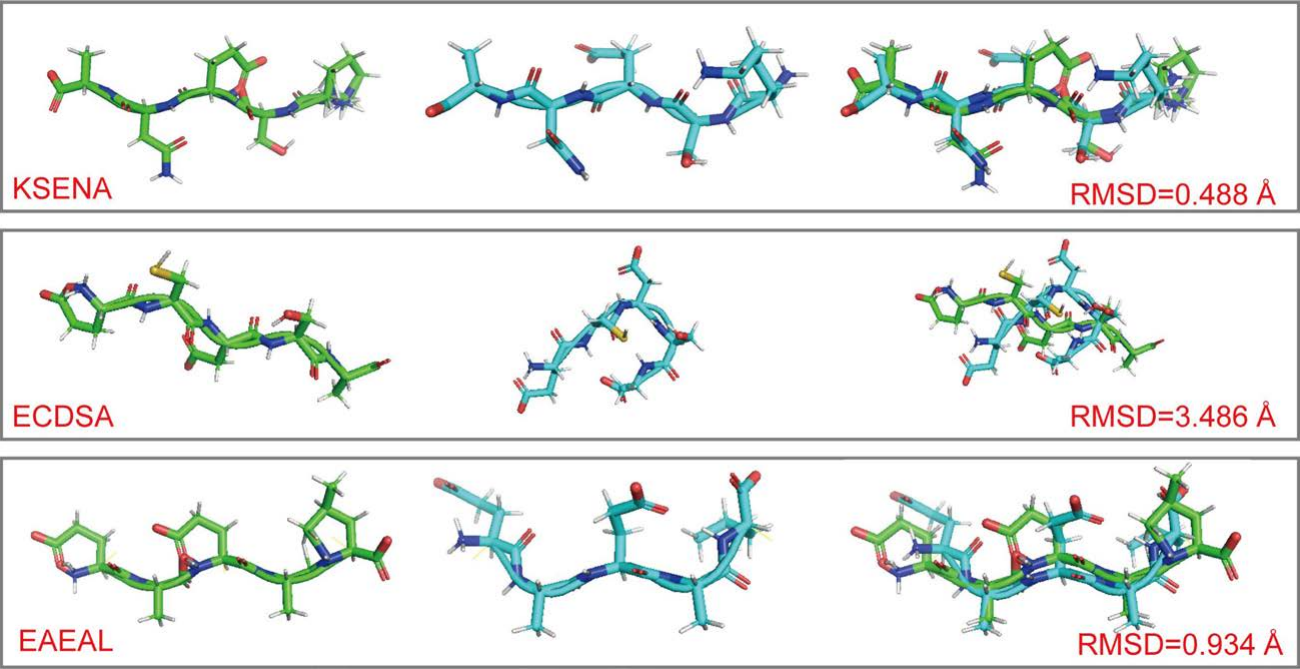
The generated peptides before and after refinement. The peptides with and without refinement were colored in cyan and green, respectively. The refinement was conducted using Rosetta minimization and CartesianMD, the resulted RMSD difference was shown in figure.

Searching the potential 3-D conformation is essential for carried out molecular docking. In this study, we adopted Rosetta Minmover and CartesianMD for optimizing the backbone and sidechains of random generated peptides prioritize to molecular docking. Firstly, Minmover was used to refine the peptide, which followed by CartesianMD to perform Monte Carlo simulation. We aim to capture the fully minimized conformation of the built peptides, and we applied 20 ps of simulation using CartesianMD. The conformation of the simulated peptides were dramatically changed by applying the CartesianMD (Fig. 3). The RMSD changes of the random selected 4 peptides varied from 0.488 to 3.486 Å (Fig. 3), indicating that our protocol can be used to capture refined peptide conformation. These energy minimized peptides were further adopted for molecular docking. Random 5-mer peptide can have 5^20^ possible compositions. In this study, we initially generated 50,000 random peptides and optimized its 3-D structure (Data S1, Supplemental Digital Content, http://links.lww.com/MD/K882).

### 3.2. Molecular docking

**Figure 4.**
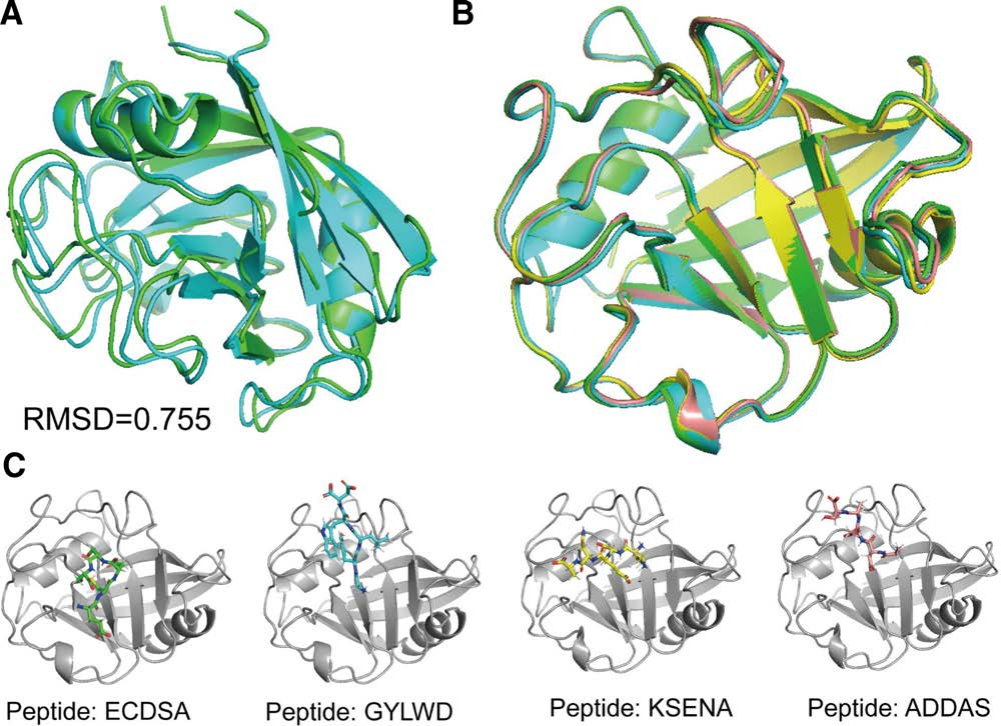
Molecular docking of CypD with different peptides. (A) The structure of CypD (PDB ID: 6R8O) before (green) and after refinement (cyan); (B) structural alignment of CypD in different docking poses; (C) the relative position of different peptides docked into CypD.

The structure of CypD was refined using 2 rounds of Rosetta relax (Table 1), and the Rosetta score of the input structure and refined structure were −576.233 Rosetta Energy Units (REU) and −749.375 REU, respectively, indicating the structural refinement is necessary for obtaining fully energy minimized structure. The RMSD differences between native and refined structure was 0.755 (Fig. 4A). Previous study highlighted the interactions between R97, Q105, S123, G114, T115, S123, N144, T149, and H169 with its native binding partner were crucial for stabilizing the binding complex (Fig. 1). In this study, the center of the indicated binding residues in CypD were selected for accommodating generated peptides. We adopted Rosetta FlexPepDock for simulating the interaction between CypD and our generated peptides. In terms of high affinity protein–ligand binding with a much stable interface, which contributed to the low overall energy of protein–peptide complex. Therefore, we used InterfaceAnalyzerMover to analyze the binding energy between protein and peptide. The dG_cross value calculated using InterfaceAnalyzerMover represents the binding energy of the interface, which we adopted for evaluating the binding affinity of protein and peptides.

**Table 1:** The Rosetta score obtained from 2 rounds of Rosetta relax on CypD.

	1st round	2nd round
nstruct_1	−606.965	−722.406
nstruct_2	−612.619	−741.954
nstruct_3	−608.452	−731.864
nstruct_4	−605.857	−721.224
nstruct_5	−605.494	−727.511
nstruct_6	−613.772	−734.773
nstruct_7	−609.597	−731.946
nstruct_8	−614.154	−730.283
nstruct_9	−607.975	−736.054
nstruct_10	−610.517	−737.08
nstruct_11	−607.928	−722.867
nstruct_12	−613.824	−733.411
nstruct_13	−606.298	−728.662
nstruct_14	−610.336	−745.398
nstruct_15	−614.302	−744.815
nstruct_16	−614.957	−748.788
nstruct_17	−605.825	−740.339
nstruct_18	−608.806	−736.602
nstruct_19	−607.702	−742.882
nstruct_20	−607.025	−732.744
nstruct_21	−605.77	−723.262
nstruct_22	−607.653	−734.859
nstruct_23	−608.210	−738.904
nstruct_24	−612.212	−741.327
nstruct_25	−608.757	−730.232
nstruct_26	−609.193	−733.86
nstruct_27	−610.445	−729.68
nstruct_28	−607.330	−730.981
nstruct_29	−610.99	−730.766
nstruct_30	−614.079	−729.536
nstruct_31	−606.106	−735.928
nstruct_32	−613.379	−731.389
nstruct_33	−609.661	−731.986
nstruct_34	−605.347	−731.41
nstruct_35	−605.063	−749.375
nstruct_36	−613.108	−749.178
nstruct_37	−613.790	−748.387
nstruct_38	−614.801	−742.587
nstruct_39	−607.114	−722.921
nstruct_40	−614.098	−722.67
nstruct_41	−606.88	−740.582
nstruct_42	−607.322	−741.642
nstruct_43	−607.012	−743.756
nstruct_44	−612.018	−743.62
nstruct_45	−614.752	−733.458
nstruct_46	−613.800	−733.402
nstruct_47	−608.751	−734.757
nstruct_48	−610.346	−739.934
nstruct_49	−611.454	−736.758
nstruct_50	−606.203	−730.191

1st = first; 2nd = second.

Our result showed that different peptide bind with CypD have very distinct Rosetta dG_cross score, which varied from −36 to 5 REU, and the complex total score varied from −668 to −546 REU (Data S1, Supplemental Digital Content, http://links.lww.com/MD/K882). The binding poses were visualized, showing that the conformation of CypD in different binding complex with minor RMSD differences (Fig. 4B). By contrast, the docked peptides in different binding complex varied obviously, indicating the docking protocol can significantly change the position of the docked peptide to search the optimized binding pose (Fig. 4C). Meanwhile, we showed that adopting the refine protocol for molecular docking, the docked peptides were not moved out from the binding pocket (Fig. 4C).

### 3.3. Virtual iterative mutagenesis for selecting the best peptides

**Figure 5.**
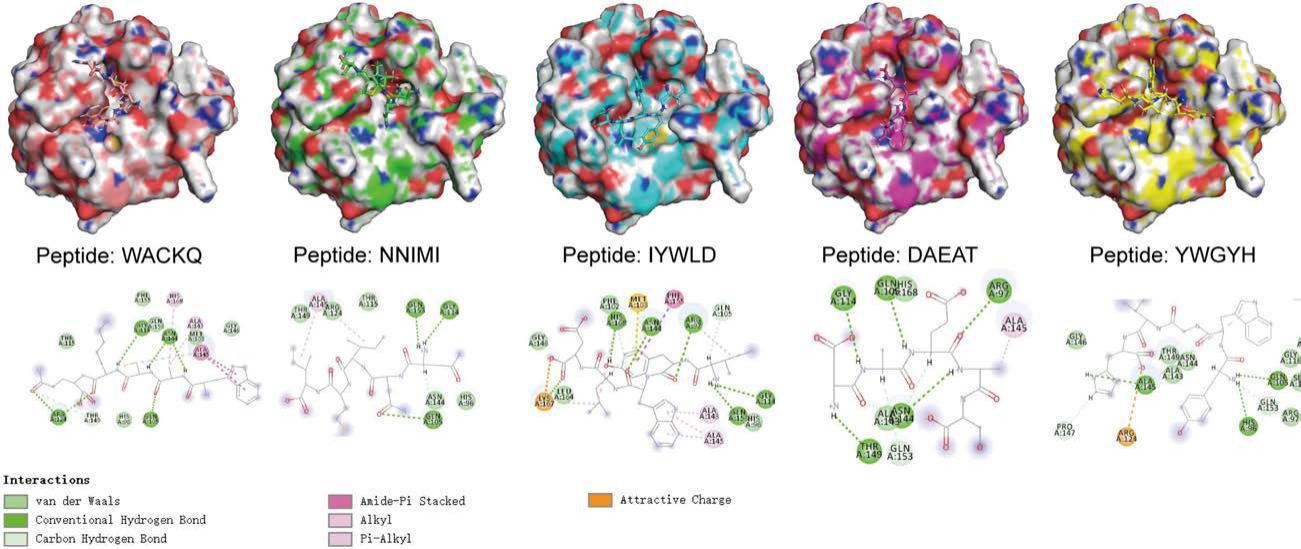
Align of thrombin-peptide complexes before and after docking. (A) Thrombin docked with peptide AMHTCLE; (B) thrombin docked with peptide IDAVLPL. Thrombin colored in cyan and green indicated structure before and after docking, while peptide colored in blue and red indicated structure before and after docking. Left: the relative position of protein and peptide; right: the 2-D view of protein–ligand interaction after docking; bottom: the types of bonds of the 2-D interactions.

The interactions between CypD and its native binder (as indicated by JV2 in PDB ID: 6r8o) were analyzed using the interfaces module of PDBePISA (https://www.ebi.ac.uk/pdbe/pisa/, accessed on October 21, 2023). The results indicated that the hydrogen bond interaction was mainly adopted by CypD-JV2 complex, and these residues including R97, Q105, R124, and N144 were key for the binding (Table 2). In the first round of molecular docking, the interactions between CypD and the top 5 peptides with the lowest dG_cross score were visualized as Figure 5, and hydrogen bonds were key for capturing docked peptides, while residues including Q105, R124, G114, N144, and Q153 were key residues (Fig. 5). These results suggested CypD adopted a similar binding mechanism to against different types of binding partners. For iteration, we selected the peptide with the lowest dG_cross score for iteration. Based on the sequence of the selected peptide, we introduced single mutation to the peptide and generated another library with 1000 peptides. Molecular docking was performed using the new library, and the resulted docking poses with dG_cross score varied from −42 Kcal/mol to −17 Kcal/mol (Data S2, Supplemental Digital Content, http://links.lww.com/MD/K883), lower than that of the initial library.

**Table 2:** Hydrogen bond interactions between CypD and JV2.

Residues in CypD	Atoms in JV2	Distance (Å)
N144	N17	2.92
N144	N20	3.01
N144	O15	3.00
R97	O19	2.93
Q105	O19	2.97
R124	O34	3.60

CypD = Cyclophilin D; JV2 = 1-(((2R,3S,6R)-3-hydroxy-2,3,4,6-tetrahydro-1H-2,6-methanobenzo[c][1,5]oxazocin-8-yl)methyl)-3-(2-((R)-2-(2-(methylthio)phenyl)pyrrolidin-1-yl)-2-oxoethyl)urea.

### 3.4. Validating peptide inhibitors against CypD

**Figure 6.**
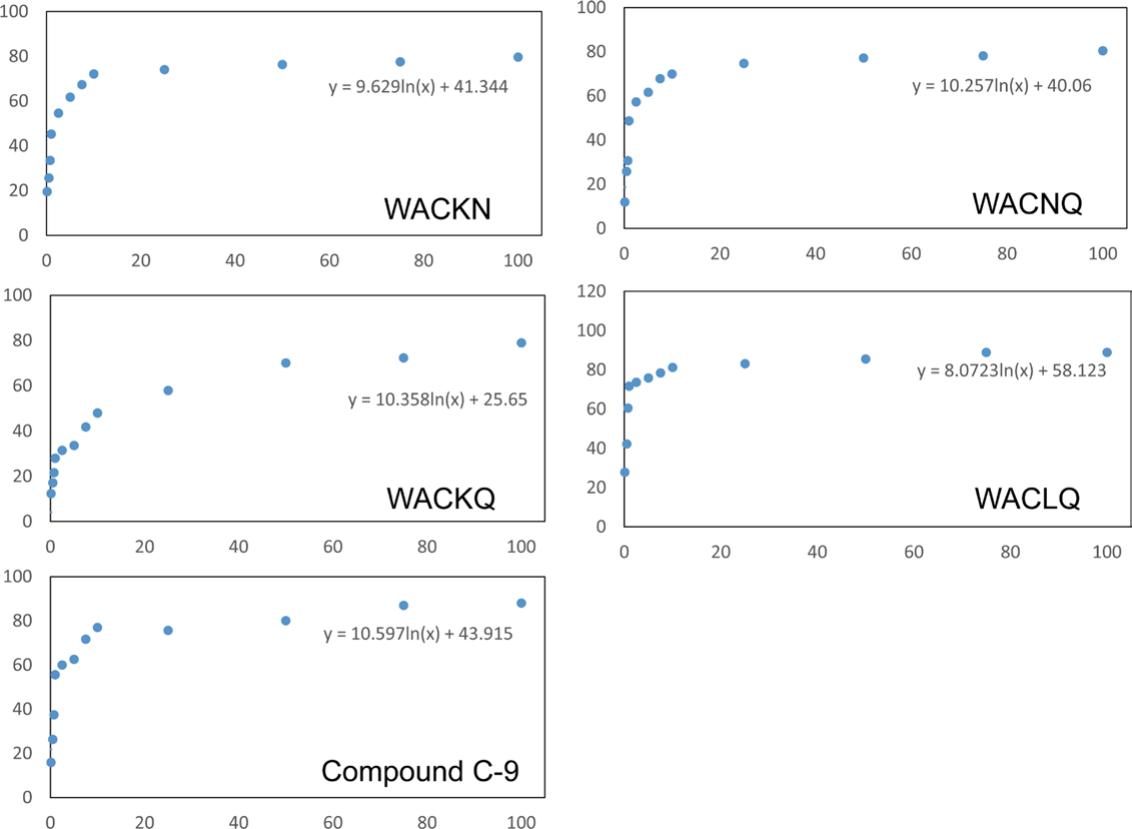
The nonlinear fitting curve for inhibitory assay. The peptide and binding compound were labeled in graph. The *x*-axis presents the peptide solution, which ranged from 0.001 to 100 μM, the *y*-axis presents the inhibitory activity (μM).

The binding affinity of selected peptides against CypD was tested using a modified inhibitory assay.^[37]^ Inhibition of CypD activity was suggested to inhibit the cyclophilin prolyl isomerization of succinate-AAPF-AMC.^[38]^ The selected 5 peptides were preincubated with CypD to validate their capability for blocking CypD activity. Regarding the selected peptides were similar, we showed 4 out of 5 selected peptides with inhibitory activity against CypD, while WACLQ with the highest affinity and CACKQ with none-activity (Table 3). Compound C-9 was previously reported as a strong binder of CypD,^[37]^ therefore, we adopted compound C-9 as a reference sample to evaluate the binding activity of the designed peptides. As shown in Table 3, the IC_50_ for compound C-9 was 1.76 μM which is minor different with previously reported 1.49 μM. The IC_50_ of WACNQ, WACLQ, WACKN, and WACKQ were 2.63, 0.37, 2.46, and 10.49 μM separately (Fig. 6). These results indicated that the *in silico*-designed peptides can be binders for CypD.

**Table 3:** Inhibitory activity of the peptides and compound C-9.

Peptide sequence	IC_50_ (μM)	dG_cross score	Total score
WACNQ	2.63	−41.99971836	−614.959
WACLQ	0.37	−41.99852588	−641.461
WACKN	2.46	−41.99826289	−643.063
WACKQ	10.49	−41.99502471	−634.548
CACKQ	–	−41.94208829	−658.253
Compound C-9	1.77	–	–

IC_50_ = half-maximal inhibitory concentration.

### 3.5. Molecular dynamics simulation

**Figure 7.**
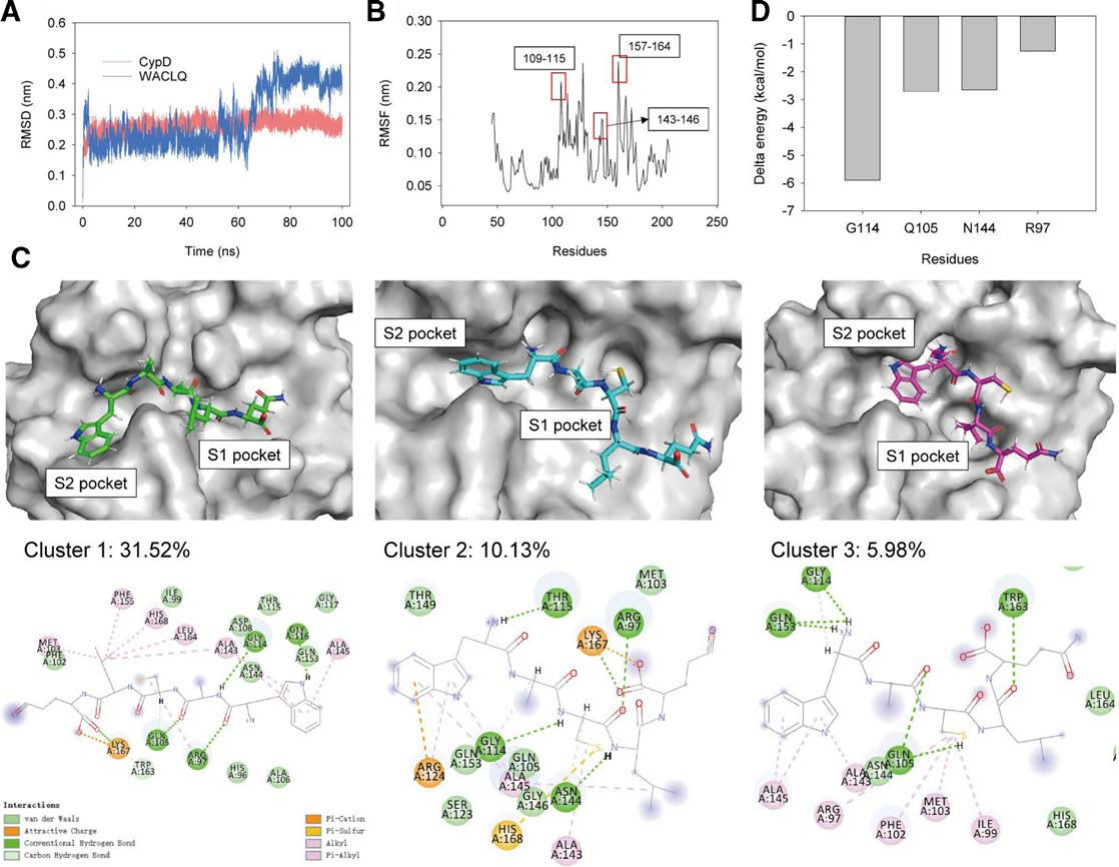
Molecular dynamics simulation of CypD binding with peptide WACLQ. RMSD (A) and RMSF (B) analysis of CypD and WACLQ; (C) RMSD-based cluster analysis of MD simulation trajectory, the portion of cluster were labeled; (D) gmxMMPBSA analysis of the interactions between CypD and WACLQ.

Molecular dynamics simulation was carried out to understand the binding mechanism between CypD and WACLQ. The docking complex of CypD with WACLQ obtained from Rosetta FlexPepDock was used for MD simulation. As shown in Figure 7A, the RMSD values both CypD and WACLQ were stabilized after 5 ns. The average RMSD for CypD and WACLQ were 2.5 Å and 1.8 Å, respectively. The stabilized trend of the peptide RMSD suggested that the binding between CypD and WACLQ can restricted the fluctuation of WACLQ. Through RMSF analysis, the 3 regions that contributed to CypD/WACLQ binding were high flexible as shown in Figure 7B. According to the RMSD values, cluster analysis was performed. The top 3 clusters of CypD/WACLQ were extracted and visualized the interactions of the interface. As shown in Figure 7C, the residues including R97, Q105, G114, and N144 were key residues for the binding between CypD and WACLQ.

Previous study demonstrated that blocking the S1 and S2 pockets in CypD can restrict its activity.^[30]^ In this study, we showed that the designed peptide WACLQ can bind to the S1 and S2 pockets of CypD (Fig. 7C). To confirm the significance of these residues, gmxMMPBSA was performed using the last 10 ns of the simulation.^[36]^ The residues including R97, Q105, G114, and N144 with higher binding affinity against WACLQ versus other residues (Fig. 7D). In addition, the contributing energy terms of the 4 key residues were analyzed and shown in Table 4, suggesting the van der Waals mainly contributed to the binding between CypD and WACLQ, and electrostatic forces showed minor contribution. As shown in Figure 7C and D, the hydrogen bonds mainly contributed to the tight binding of CypD and WACLQ, which in agree with mmPBSA analysis.

**Table 4:** Contributing energy terms of key residues for CypD and WACLQ binding.

Residue	Nonpolar solvation	Polar solvation	Electrostatic	van der Waals	Total energy (Kcal/mol)
*Last 10 ns*					
G114	−0.30	0.31	−0.11	−5.80	−5.90
Q105	−0.32	0.33	−0.10	−2.61	−2.70
N144	−0.31	0.31	−0.04	−2.60	−2.64
R97	−0.21	0.25	−0.06	−1.24	−1.26
*Before 60 ns*					
G116	−0.21	0.21	−0.07	−1.33	−1.4
Q105	−0.23	0.23	−0.09	−2.77	−2.86
G114	−0.29	0.28	−0.09	−3.12	−3.22
R97	−0.56	0.56	−0.1	−6.01	−6.11
*After 60 ns*					
R97	−0.19	0.19	−0.06	−1.01	−1.07
Q105	−0.12	0.12	−0.07	−1.84	−1.91
G114	−0.22	0.23	−0.07	−2.51	−2.57
T115	−0.23	0.23	−0.11	−2.89	−3
N144	−0.33	0.34	−0.15	−3.55	−3.69

CypD = Cyclophilin D.

The RMSD for peptide WACLQ varied dramatically before and after 60 ns (Fig. 7A), suggesting the position of peptide varied during the simulation. Extracting average binding complex structure from the 2 state revealing the structure of Cluster 1 and 2 highly corresponded to the structure before and after 60 ns, respectively (Fig. 7C). The residues including R97, Q105, G114, and G116 mainly supporting the binding between CypD and WACLQ before 60 ns, and changed to R97, Q105, G114, T115, and N144 after 60 ns. Notably, the binding force was stronger before 60 ns of simulation than that after 60 ns (Table 4). These results may support for further design of peptides for binding of thrombin.

## 4. Conclusions

In this study, we introduced computer-aided method for designing peptide binders. Through using this method, a peptide library was initially built and docked into the receptor. The peptides with strong binding capacity against the receptor was selected for virtual iterative mutagenesis. We adopted this method for designing CypD binders, and successfully obtained peptide binder with strong affinity against CypD. Our in vitro validation suggested that the designed peptide WACLQ display a higher binding affinity than the previously reported binder compound C-9. We showed our designing method is robust, which can be used for designing peptide binders for other proteins. Meanwhile, the peptide binder designed in this study may become a therapeutic strategy for AP.

## Author contributions

**Investigation:** Yuehong Li, Ting Liu, Xiaoyan Lai, Huifang Xie.

**Methodology:** Heng Tang, Shuangchan Wu.

**Writing – original draft:** Yuehong Li.

**Writing – review & editing:** Yongshun Li.

## Supplementary Material








