# Supplementary material for: Rational design peptide inhibitors of Cyclophilin D as a potential treatment for acute pancreatitis
Source: Medicine (Baltimore). 2023 Dec 1;102(48):e36188. doi: 10.1097/MD.0000000000036188 (PMC10695616; doi:10.1097/MD.0000000000036188)
Supplement: Supplementary file 1 [file medi-102-e36188-s001.docx]

**Table S1** Command line for Rosetta and Gromacs

| **##First round relax**  relax.mpi.linuxgccrelease -s input_pdb.pdb -relax:constrain_relax_to_start_coords -ramp_constraints false -relax:coord_constrain_sidechains -nstruct 50 -ex1 -ex2 -use_input_sc -flip_HNQ -no_optH false |
| --- |
| **##Second round relax**  relax.mpi.linuxgccrelease -s 1.pdb -nstruct 50 -ex1 -ex2 -use_input_sc -flip_HNQ -no_optH false  -relax:cartesian -score:weights ref2015_cart -crystal_refine |
| **##Build peptide**  BuildPeptide.mpi.linuxgccrelease –in:file:fasta peptide.fasta –out:file:o peptide.pdb |
| **##Simulating peptide backbone**  **##Rosetta script**  <ROSETTASCRIPTS>  <SCOREFXNS>  <ScoreFunction name="r2014" weights="talaris2014_cart"/>  </SCOREFXNS>  <RESIDUE_SELECTORS>  </RESIDUE_SELECTORS>  <TASKOPERATIONS>  </TASKOPERATIONS>  <FILTERS>  </FILTERS>  <MOVERS>  <MinMover name="minimize" scorefxn="r2014" chi="1" bb="1" tolerance="0.0000000001" max_iter="1000000" />  <CartesianMD name="MD" scorefxn="r2014" nstep="100000" temp="200" premin="500" />  </MOVERS>  <APPLY_TO_POSE>  </APPLY_TO_POSE>  <PROTOCOLS>  <Add mover_name="minimize"/>  <Add mover_name="MD"/>  </PROTOCOLS>  <OUTPUT />  </ROSETTASCRIPTS>  **##Command line**  rosetta_scripts.mpi.linuxgccrelease -s peptide.pdb -parser:protocol script.xml -in:file:fullatom -ignore_unrecognized_res -ex1 -ex2 -restore_talaris_behavior |
| **##Gromacs editconf**  gmx_mpi editconf -f input_ligand.pdb -center coordinate_x coordinate_y coordinate_z -o output_ligand.pdb |
