# Supplementary material for: Rational design peptide inhibitors of Cyclophilin D as a potential treatment for acute pancreatitis
Source: Medicine (Baltimore). 2023 Dec 1;102(48):e36188. doi: 10.1097/MD.0000000000036188 (PMC10695616; doi:10.1097/MD.0000000000036188)
Supplement: Supplementary file 2 [file medi-102-e36188-s002.docx]

**Table S2** Python and Rosetta script for molecular docking.

| **## Python script for generating peptide sequences**  import random  def seq_gen(generate_number=1000,mers=5):  seqs = []  residues_list = ['A', 'G', 'D', 'N', 'F', 'P', 'Q', 'C', 'E', 'H', 'I', 'K', 'L', 'M', 'R', 'S', 'T', 'V', 'W', 'Y']  for i in range(generate_number):  a =(random.choices(residues_list,k=mers))  seq = ''  seq = seq.join(a)  seqs.append(seq)  for i in seqs:  f = open('%s.fasta'%i,'w')  f.write('%s'%i)  f.close()  f = open('buildpep.sh','a+')  for i in seqs:  f.write('BuildPeptide.mpi.linuxgccrelease -in:file:fasta %s.fasta -out:file:o %s.pdb\n'%(i,i))  f.close() |
| --- |
| **## Python script for iterative mutagenesis**  import random  def seq_gen(generate_number=1000,pepseq='WACKQ'):  seqs = []  residues_list = ['A', 'G', 'D', 'N', 'F', 'P', 'Q', 'C', 'E', 'H', 'I', 'K', 'L', 'M', 'R', 'S', 'T', 'V', 'W', 'Y']  for i in range(generate_number):  a =(random.choice(residues_list))  r = int(random.uniform(0,5))  a = pepseq[0:r]+a+pepseq[(r+1):5]  seqs.append(a)  f = open('newpep.txt' , 'a+')  for i in seqs:  f.write('%s\n'%i)  f.close() |
| **## Python script for renaming PDB chain**  import sys  pdbdata = open('1.pdb','r').readlines()  chain = 'B'  output = open('1.pdb','w')  for line in pdbdata:  if line[0:4] =='ATOM':  try:  newline = '%s%s%s'%(line[0:21],chain,line[22:])  output.write(newline)  except:  print('bad') |
| **## Rosetta Script: dock**  <ROSETTASCRIPTS>  <SCOREFXNS>  <ScoreFunction name="r2015" weights="ref2015" />  </SCOREFXNS>    <RESIDUE_SELECTORS>  </RESIDUE_SELECTORS>  <TASKOPERATIONS>  <RestrictToRepacking name="no_design" />  </TASKOPERATIONS>  <FILTERS>  </FILTERS>    <MOVERS>  <FlexPepDock name="dock" pep_refine="1" />  <InterfaceAnalyzerMover name="dg" scorefxn="r2015" packstat="1" pack_input="0" jump="1" tracer="0" use_jobname="1" resfile="0" />  <FastRelax name="fastrelax" scorefxn="r2015" task_operations="no_design" relaxscript="InterfaceRelax2019" >  <MoveMap name="only_move_protein" >  <Chain number="1" chi="1" bb="1" />  </MoveMap>  </FastRelax>  <MinMover name="min_mover" scorefxn="r2015" tolerance="0.000001" bb="1" chi="1" jump="1" max_iter="5000" />  </MOVERS>  <APPLY_TO_POSE>  </APPLY_TO_POSE>  <PROTOCOLS>  <Add mover="dock" />  <Add mover="min_mover"/>  <Add mover="fastrelax" />  <Add mover="dg" />  </PROTOCOLS>  <OUTPUT />  </ROSETTASCRIPTS> |
| **##Command line**  rosetta_scripts.mpi.linuxgccrelease -s protein_peptide_complex.pdb -parser:protocol run.xml -in:file:fullatom -ignore_unrecognized_res -ex1 -ex2 -overwrite |
